# Matched Whole-Genome Sequencing (WGS) and Whole-Exome Sequencing (WES) of Tumor Tissue with Circulating Tumor DNA (ctDNA) Analysis: Complementary Modalities in Clinical Practice

**DOI:** 10.3390/cancers11091399

**Published:** 2019-09-19

**Authors:** Robin Imperial, Marjan Nazer, Zaheer Ahmed, Audrey E. Kam, Timothy J. Pluard, Waled Bahaj, Mia Levy, Timothy M. Kuzel, Dana M. Hayden, Sam G. Pappas, Janakiraman Subramanian, Ashiq Masood

**Affiliations:** 1 Department of Medicine, Kansas City School of Medicine, University of Missouri, Kansas City, MO 64110, USA; imperialr@umkc.edu (R.I.); nazerm@umkc.edu (M.N.); ahmedza@umkc.edu (Z.A.); tpluard@saint-lukes.org (T.J.P.); bahajw@umkc.edu (W.B.); jsubramanian@saint-lukes.org (J.S.); 2 Division of Hematology/Oncology and Cell Therapy, Rush University Medical Center, Chicago, IL 60612, USA; Audrey_E_Kam@rush.edu (A.E.K.); Mia_Levy@rush.edu (M.L.); Timothy_Kuzel@rush.edu (T.M.K.); 3 Division of Oncology, Saint Luke’s Cancer Institute, Kansas City, MO 64111, USA; 4 Rush Precision Oncology Program, Rush University Medical Center, Chicago, IL 60612, USA; 5 Division of Surgical Oncology, Rush University Medical Center, Chicago, IL 60612, USA; Dana_M_Hayden@rush.edu (D.M.H.); Sam_G_Pappas@rush.edu (S.G.P.)

**Keywords:** circulating tumor DNA, next generation sequencing, driver alterations, actionable alterations, concordance

## Abstract

Tumor heterogeneity, especially intratumoral heterogeneity, is a primary reason for treatment failure. A single biopsy may not reflect the complete genomic architecture of the tumor needed to make therapeutic decisions. Circulating tumor DNA (ctDNA) is believed to overcome these limitations. We analyzed concordance between ctDNA and whole-exome sequencing/whole-genome sequencing (WES/WGS) of tumor samples from patients with breast (*n* = 12), gastrointestinal (*n* = 20), lung (*n* = 19), and other tumor types (*n* = 13). Correlation in the driver, hotspot, and actionable alterations was studied. Three cases in which more-in-depth genomic analysis was required have been presented. A total 58% (37/64) of patients had at least one concordant mutation. Patients who had received systemic therapy before tissue next-generation sequencing (NGS) and ctDNA analysis showed high concordance (78% (21/27) vs. 43% (12/28) *p* = 0.01, respectively). Obtaining both NGS and ctDNA increased actionable alterations from 28% (18/64) to 52% (33/64) in our patients. Twenty-one patients had mutually exclusive actionable alterations seen only in either tissue NGS or ctDNA samples. Somatic hotspot mutation analysis showed significant discordance between tissue NGS and ctDNA analysis, denoting significant tumor heterogeneity in these malignancies. Increased tissue tumor mutation burden (TMB) positively correlated with the number of ctDNA mutations in patients who had received systemic therapy, but not in treatment-naïve patients. Prior systemic therapy and TMB may affect concordance and should be taken into consideration in future studies. Incorporating driver, actionable, and hotspot analysis may help to further refine the correlation between these two platforms. Tissue NGS and ctDNA are complimentary, and if done in conjunction, may increase the detection rate of actionable alterations and potentially therapeutic targets.

## 1. Introduction

The increasing availability of next-generation sequencing (NGS) coupled with the identification and targeting of individual oncogenic drivers has generated great interest in genomic-driven therapies. Several genomic markers are routinely used to guide therapeutic decisions in multiple solid tumors. Examples include epidermal growth factor receptor (EGFR) inhibitors, such as erlotinib in advanced lung adenocarcinoma, and cetuximab and panitumumab in metastatic colorectal cancers. Furthermore, recent prospective studies have shown improved survival when patients are treated with genomic-driven therapies [1,2]. Traditionally, genomic-driven therapeutic decisions have been based primarily on tissue NGS. However, tissue heterogeneity, intra-tumoral heterogeneity (heterogeneity among the tumor cells), inter-metastatic heterogeneity (heterogeneity among different metastatic lesions in the same patient), and temporal heterogeneity (heterogeneity during tumor evolution) often exist and, if not taken into consideration, may lead to missed opportunities for targeted therapies or inappropriate therapeutic interventions. While obtaining multiple biopsies from tumor sites seems like an attractive solution, logistical and safety limitations make it impractical in a clinical setting.

Circulating tumor DNA (ctDNA) is becoming more commonly used as a surrogate for tumor-tissue-based genomic analysis [3]. ctDNA is shed by tumor cells during apoptosis and necrosis in the peripheral blood. It is a less invasive test that can detect genomic alterations including point mutations, rearrangements, amplifications, and aneuploidy. ctDNA has the potential to reveal the complete molecular architecture of all tumor clones and subclones, and therefore depicts the tumor’s dynamic evolution and its current mutational landscape. In addition, ctDNA can be used to monitor therapeutic response [4,5,6] and acquired resistance following therapeutic interventions [7,8]. As such, several clinical trials based on somatic alterations in ctDNA are currently underway. A large prospective basket/umbrella trial, Targeted Agent and Profiling Utilization Registry (TAPUR), sponsored by the American Society of Clinical Oncology (ASCO), is enrolling patients based on ctDNA (NCT02693535). Another prospective study to further delineate the role of ctDNA in solid tumors is ongoing in Korea [9].

Multiple groups, including ours, have previously described concordance between tissue biopsy and ctDNA using targeted tissue NGS gene panels, which include only a few hundred genes [10,11,12,13,14]. However, to the best of our knowledge, concordance using tissue whole-genome (WGS) and whole-exome sequencing (WES) platforms and ctDNA gene panel has not been described.

Tumor mutational burden (TMB, mutations/megabase) has recently become an area of interest, as high TMB is associated with improved response to immunecheckpoint inhibitor therapies [15]. While the gold standard for measuring TMB is WES/WGS, targeted sequencing may provide a more feasible method in a clinical setting, and has shown marked variations between TMB cutoffs and survival in various tumor types [16]. Furthermore, ctDNA mutation load is being investigated as a potential surrogate. However, the correlation between these platforms has yet to be elucidated, thereby making it difficult to determine a cutoff value for which immunotherapy should be implemented in clinical practice.

To address these issues, we performed a real-world oncology retrospective analysis of common solid tumors including breast, gastrointestinal, and lung cancers, examining the concordance between tissue WES/WGS and ctDNA platforms. Similar to previous studies, our comprehensive analysis was conducted at the patient and gene levels. It included the evaluation of potential driver mutations and known actionable mutations [17,18]. Unique to our study, we explored the concordance of hotspot mutations. These missense mutations are believed to have the potential to disrupt protein functional domains leading to tumorigenesis and clonal evolution [19].

We also investigated the various factors which may have influenced the concordance and discordance in our samples, including timing between biopsies, site of biopsy, number of metastatic sites, administration of chemotherapy, tumor mutational burden, and cancer type.

## 2. Materials and Methods

### Methods

The Institutional Review Board (IRB) of the Saint Luke’s Health System approved this study. Written informed consent was waived as per the IRB. The study was conducted in accordance with the Declaration of Helsinki. Tumor samples sent for WES/WGS were prepared from formalin-fixed, paraffin-embedded surgical tissue specimens from either primary tumors or metastatic sites. As this study was conducted in real-world clinical scenarios, commercially available platforms were utilized. Quality control of sequencing and methodology including read depth threshold, variant allele frequency(VAF), and variation calling pipeline was subjected to specifications as determined by each respective platform (see Supplementary Materials). However, specifics regarding VAF and other thresholds were not included in the clinical reports by the various commercial platforms. WGS and WES were carried out on the NantOmics and Tempus platforms, respectively, using tumor tissue and matched peripheral blood. Peripheral blood for ctDNA was analyzed using the Circulogene and Guardant platforms. The Circulogene targeted NGS assay tested for 50 genes, whereas Guardant tested for 73 genes (Supplementary Table S1). All platforms included analyses of single nucleotide variations (SNV), copy number alterations, amplifications, insertions, deletions, and frameshift mutations.

Concordance and discordance were described similarly to our and other previous studies [10,13,20]. Concordance was defined as having at least one same specific gene and amino acid single nucleotide variation (SNV), insertion or deletion (INDEL), fusion, or copy number alteration in both matched tumor and ctDNA samples. For example, a *PIK3CA* R1047L somatic alteration detected in both tissue NGS and matched ctDNA would be classified as a positive concordance. In contrast, a *PIK3CA* R1047L mutation in tissue NGS and *PIK3CA* N354K mutation in matched ctDNA would be classified as discordant.

Actionable and driver alterations analyses were performed by cross-referencing all discovered somatic alterations in our cohort from tissue NGS and ctDNA analysis with recently published large pan-cancer studies [17,18]. In addition, we also studied hotspot oncogenic and tumor suppressor somatic alterations using recently published studies [19]. Tumor mutational burden (TMB) in tumor tissue was provided in clinical reports by the commercially available NantOmics and Tempus platforms using their bioinformatics pipelines, and was correlated with a number of matched somatic ctDNA mutations using the Pearson correlation coefficient.

Descriptive statistical analysis was used to summarize the somatic genomic alterations discovered in this study. When appropriate, statistical analysis was carried out using Fisher’s exact test, chi-square analysis, and two-sample *t*-test. 

## 3. Results

### 3.1. Patient Characteristics

A total of 64 patients with advanced or metastatic cancers were analyzed using matched tumor WES/WGS and ctDNA samples (Table 1, Supplementary Table S2). Our analysis included solid tumors: breast (*n* = 12), gastrointestinal (GI) (*n* = 20), lung (*n* = 19), and other (*n* = 13). Tumor samples consisted of mostly stage IV tumors and a few stage III tumors (Table 1). A total 59% (*n* = 38) of tumor biopsies were taken from the primary tumor site, while 41% (*n* = 26) were taken from metastatic sites.

Most patients received chemotherapies, while some received immunotherapies and targeted agents depending on tumor histology and molecular profile (Supplementary Table S3). All other therapies have been referred to as systemic therapies in this manuscript.

### 3.2. Patient-Level Analysis

At the patient level, 58% (37/64) of patients had at least one concordant alteration, the most common of which involved the *KRAS* G12 locus (eight mutations: *KRAS* G12C/D/V). We observed high concordance in metastatic breast cancers and to a lesser extent in metastatic lung cancers (83% (10/12) and 68% (13/19), respectively). Concordance was higher when patients had received systemic therapies (for therapies see Supplementary Table S3) prior to either tissue biopsy or ctDNA testing compared to treatment-naïve patients (78% (21/27) vs. 43% (12/28) respectively, *p* = 0.01). All breast cancer patients (12/12) had received systemic therapies prior to both tissue NGS and ctDNA (Table 2), and 10/16 lung cancer patients had received systemic therapy prior to tissue NGS and ctDNA analysis, whereas only 3/20 GI cancer patients had received systemic treatment prior to tissue and ctDNA biopsies, and 13 patients were treatment-naïve. Concordance among treated lung and GI cancers was 80% (8/10, *p* = 0.058) and 33% (1/3, *p* = 0.56), respectively. In the treatment-naïve lung and GI cancer subgroups, concordance was 50% (3/6) and 54% (7/13, *p* = 0.77), respectively.

Interestingly, we found other factors that may have influenced patient-level concordance. Concordance was significantly higher when TMB was ≥ 2, compared to TMB < 2 (69% (27/39) vs. 36% (8/22), respectively; *p* = 0.013). We also observed that biopsies from metastatic sites tended to have higher concordance compared to primary sites (64% (25/29) vs. 48% (12/25); *p* = 0.20). However, when analyzed by cancer type, no appreciable difference was observed between primary and metastatic tumor NGS (Table 2).

We did not observe a significant difference in patient-level concordance temporally and spatially. The concordance of any detected mutation was 55% (24/44) compared to 65% (13/20) when the time interval between tissue NGS and ctDNA analysis was more or less than 90 days, respectively (*p* = 0.43). Virtually no difference was seen with a patient-level concordance of driver mutations before or after 90 days (52% (23/44) vs. 60% (12/20), respectively; *p* = 0.60). However, targetable alterations tended to have higher positive concordance when the time between tissue NGS and ctDNA was longer (<90 days 32% (9/28) vs. ≥90 days 63% (10/16); *p* = 0.065; (Table 2)).

Given that the number of metastatic sites at the time of first biopsy may also have contributed to the concordance between tissue NGS and ctDNA, we compared the concordance of matched samples with one metastatic site at the time of tissue NGS to those with two or more sites. We found no significant difference either in our entire cohort or within each cancer subtype (Table 2).

### 3.3. Gene-Level, Driver Mutation, and Actionable Alteration Analysis

The average number of mutations per ctDNA sample was 4.48 (range 0–20). The average number of mutations per tumor sample was 82.25 (range 1–425). Analysis by tumor type revealed that GI tumors had the lowest average number of mutations per tissue sample (34.05 mutations), whereas lung tumors had the highest number (123.16 mutations). The mutational burdens of our datasets, especially GI cancers, were lower than historical TCGA studies. The reasons might include a different patient population and the stringent bioinformatics pipeline employed in CLIA-certified clinical sequencing to report only confident calls able to be used to make therapeutic decisions in a clinical setting [21,22,23,24,25,26,27,28,29,30,31,32].

At the gene level, our analysis revealed high discordance among all tissue NGS and matched ctDNA samples. Concordance in all 64 samples was only 16% (60 concordant events in 374 mutations), and we did not observe a significant difference in gene-level concordance between cancer types (Table 2). However, gene-level concordance of GI cancers appeared to the lowest among the three cancer types (GI 15%, breast 20%, lung 22%).

The difference in concordance observed between the treatment-naïve and treated samples seen at the patient level was also reflected at the gene level. Gene-level concordance was significantly higher when systemic therapy was given prior to any NGS (21% vs. 11%, respectively; *p* = 0.014).

Given that all breast cancer patients in this study were given treatment prior to tissue NGS and ctDNA analysis, we looked at the gene-level concordance of the remaining cohort excluding breast cancers. Our cohort excluding breast cancers had similar gene-level positive and negative concordance when comparing treated to treatment-naïve patients (21% vs. 11%, respectively; *p* = 0.030).

Lung cancer gene-level concordance in treated patients was 26% compared to 11% in treatment-naïve patients. In contrast, this pattern appeared to be reversed in GI malignancies; gene-level positive concordance with treated patients was 7.1% compared to 16% in treatment-naïve patients (*p* = 0.41).

**Figure 1:**
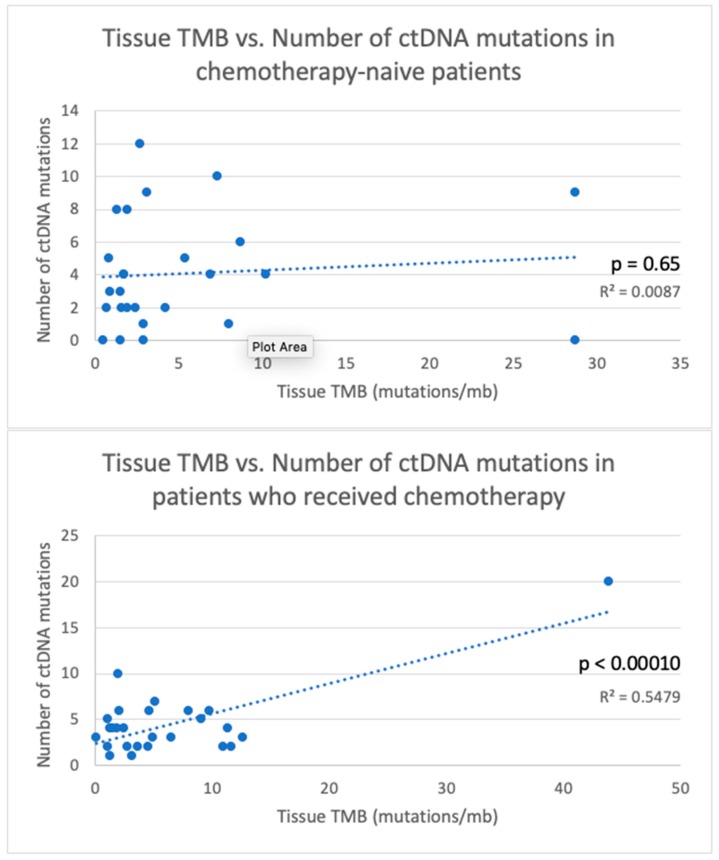
Scatter plots showing the relationships between tissue tumor mutation burden (TMB, mut/mb) and the number of ctDNA mutations. TMB was positively correlated with the number of ctDNA mutations only in those who received chemotherapy prior to tissue next generation sequencing or ctDNA analysis.

In examining the relationship of TMB and ctDNA mutations, we found that tissue NGS positively correlated with the number of ctDNA mutations when looking broadly at our entire cohort (Pearson r(62) = +0.48, *p* < 0.0010). However, this correlation was largely dependent on the strength of those who had received systemic therapy prior to any NGS (Pearson r(25) = +0.74, *p* < 0.00010). Treatment-naïve patients did not have any appreciable correlation with TMB or number of ctDNA mutations (Pearson r(27) = +0.11, *p* = 0.60) (Figure 1). When we looked at correlation based on tumor type, we found a significant correlation between tissue TMB and the number of ctDNA mutations among breast cancer patients, but not in lung or GI cancer patients (Supplementary Figure S1, Supplementary Table S4). As mentioned previously, all breast cancer patients had received prior chemotherapy. In lung and GI cancer patients, we did not have a large enough sample size to analyze TMB and number of ctDNA mutations in relation to chemotherapy.

Similar to our patient-level analysis, gene-level analysis did not reveal a statistical difference with regard to tissue site for tissue NGS (primary 14% vs. metastasis 17%, *p* = 0.5036) or time course (<90 days 16% vs. ≥90 days 18%, *p* = 0.7052). However, there was a significant correlation between increased number of metastases and gene-level concordance (Pearson r(55) = +0.34, *p* = 0.010).

With regard to driver alterations, the yield was significantly increased when tissue NGS was combined with ctDNA. Overall, 671 driver alterations were identified (tissue NGS 406, ctDNA 203, concordant 62) (Supplementary Figure S2). In the subgroup analysis, lung cancers identified 183 driver alterations (tissue NGS: 114; ctDNA: 47; concordant: 22 (12%)). Breast cancers identified 172 driver alterations (tissue NGS: 108; ctDNA: 47; concordant: 17 (10%)). GI cancers identified 150 driver alterations (tissue NGS: 68; ctDNA: 64; concordant: 18 (12%)).

Receipt of prior systemic therapy did not significantly affect the concordance of driver alterations: 9.4% in treated samples compared to 7.1% in treatment-naïve samples, respectively. Similarly, TMB also did not affect the positive concordance of driver alterations (TMB <2: 9.8% vs. TMB ≥2: 8.8%).

We found 62 actionable alterations in 33 patients. 13/62 actionable mutations were present only in tissue NGS, while 35/62 actionable mutations were present only in ctDNA. A total 14/62 mutations were concordant between tissue NGS and matched ctDNA, seen in 12 patients. Thus, 21 patients had mutually exclusive targetable alterations seen only in either tissue NGS or ctDNA samples (Supplementary Figure S2).

### 3.4. Hotspot Mutational Analysis Concordance

A total 13 patients in our cohort did not have any detected hotspot mutations. Among the remaining 51 patients, we discovered 91 total hotspot mutations in 17 genes. Of these hotspots, 56 were unique, with the majority of them occurring in *TP53* (30/56) (Table 3, Supplementary Figure S3A, Supplementary Table S5a,b). A total 34% (31/91) of all hotspots were concordant.

The most common genes with concordant hotspots were *TP53* (14), *KRAS* (10), *PIK3CA* (2) and *BRAF* (2) (Table 4). Although *TP53* had the most concordant hotspots, no single hotspot had a more notable frequency (all ≤ 2/14, Supplementary Figure S3B). Similarly, *TP53* had the most discordant hotspots (*n* = 34: 15 tumor-only, 19 ctDNA-only). The discordant hotspots were also relatively spread out without significant frequency differences (all ≤ 3/34). Hotspot concordance among breast, GI, and lung cancer was 26% (5/19), 33% (11/33), and 43% (12/28), respectively. *KRAS* G12 was the most concordant hotspot (8/31) in all tumor types. However, it was also present among discordant tumor hotspots (5/29). Of the non-breast cancer patients, 13 out of 52 had *KRAS* G12 hotspot alterations. Furthermore, *KRAS* G13 alterations were present in two lung cancer patients. It was notably absent from discordant ctDNA hotspots (0/31) suggesting that a truncal mutational event is essential for early tumorigenesis.

*KRAS* G12 was the most concordant hotspot among GI cancers (4/20 patients). It was also the most discordant mutation (6/20 patients: 4 tumor-only, 2 ctDNA only). Similarly, in lung cancer samples, *KRAS* G12 was the most concordant hotspot (4/12). Breast cancers appeared to be driven primarily by *TP53* (8/14 total breast hotspots) and *PIK3CA* (3/14 total breast hotspots) mutations, which is in agreement with large tissue-NGS studies [33].

Consistent with our analysis of overall mutations, positive concordance of hotspot alterations may have been influenced by the administration of systemic therapy prior to tissue NGS or ctDNA analysis. In treated patients, hotspot concordance was 43% (15/35) compared to 24% (11/46) in treatment-naïve patients (Table 4). Specifically, we found *KRAS* G12/G13 hotspots to have a statistically significant concordance in treated patients vs. treatment-naïve patients (100% (5/5) vs. 33% (3/9); *p* = 0.031).

We did not find a significant effect of TMB and timing on hotspot concordance (TMB Pearson r(48) = −0.078; timing <90 days 32% vs. ≥90 days 38%), suggesting that these mutations are essential for tumor initiation and progression in all tumor types, irrespective of their mutator phenotype.

### 3.5. Patient Examples

**Figure 2:**
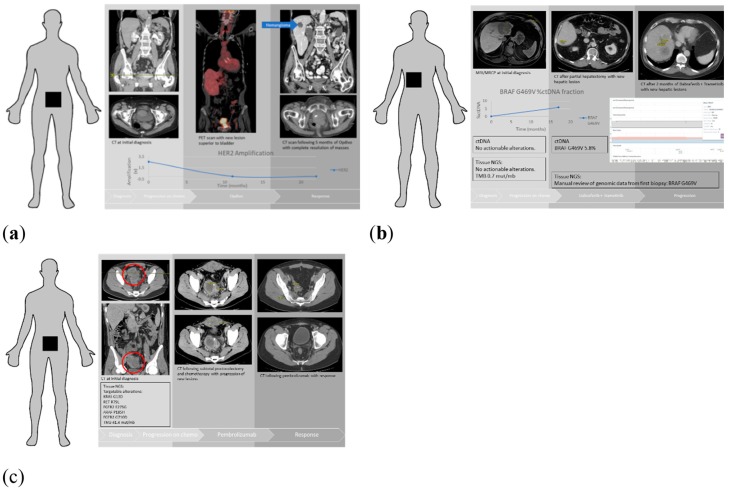
Patient example clinical course. (**a**) Patient A had progression of squamous cell bladder carcinoma, with a new lesion caudal to the bladder identified by positron emission tomography (PET) scan while on standard of care chemotherapy. Tissue next generation sequencing (tissue NGS) identified *HER-2* amplification on manual review. Patient started on Opdivo with significant response and reduction of *HER-2* amplification to undetectable levels. (**b**) Patient B had progression of cholangiocarcinoma on standard of care chemotherapy. No targetable alterations were seen on initial tissue NGS. Follow up ctDNA identified *BRAF* G469V as a possible targetable alteration. The initial tissue NGS was manually reviewed and *BRAF* G469V was also present. Patient B was started on dabrafenib and trametinib but unfortunately progressed. (**c**) Patient C had progression of colon cancer following subtotal proctocolectomy and standard-of-care chemotherapy. Multiple targetable alterations were identified including members of the RTK/RAS/MAPK pathway. Patient C was started on pembrolizumab with a dramatic response.

Patient A is an 85 year-old who was diagnosed with locally advanced small cell carcinoma of the bladder (Figure 2a). This patient was treated with carboplatin and etoposide, and progressed after three cycles with the development of a new pelvic mass and bone metastases. WGS showed a high tumor mutational burden of 10.2 mutations/Mb with no actionable alterations. ctDNA showed *HER-2* amplification. However, on manual review of tissue NGS BAM files, *HER-2* showed 4.6× copy number amplification, and proteomics also showed high *HER-2* expression in tissue NGS. Patient A was treated with nivolumab and had a dramatic response to therapy. Repeat imaging showed complete response and repeated ctDNA showed resolution of her *HER-2* amplification.

Patient B is a 79 year-old with intrahepatic cholangiocarcinoma (Stage IVA (pT2bN1)), who was treated with gemcitabine and capecitabine for six months after partial hepatectomy (Figure 2b). A year later, patient developed numerous liver metastases and was subsequently treated with FOLFOX. Tissue NGS from partial hepatectomy specimen and ctDNA after initial surgery reported no actionable alterations. The ctDNA on recurrence revealed a *BRAF* G469V mutation. On manual review of his tissue NGS BAM file, *BRAF* G469V was noted to be present, which was not detected by the variant annotation algorithm, likely due to being a subclonal event. The patient progressed on FOLFOX and was then started on trametinib and dabrafenib. Unfortunately, the patient then progressed on trametinib and dabrafenib, which reinforces the fact that drugs employed in *BRAF* V600E-mutated patients do not apply to other variants of *BRAF* mutations. This case also demonstrates the limitations of precision medicine and the need for functional assays or organoid models to develop effective drug screens.

Patient C is a 29 year-old with Lynch syndrome and stage III colon cancer who underwent total colectomy (Figure 2c). While receiving adjuvant systemic therapy, patient was found to have new metastases in the abdomen and pelvis. WGS revealed a hypermutated phenotype with 41.4 mutations/Mb. Somatic mutations results returned targetable alterations in *RET* R79L, *FGFR2* E275G, *ARAF* P185H, *FGFR3* G710D. These are not likely to be actionable alterations given that mutations in the Receptor Tyrosine Kinase/Ras GTPase/MAP kinase (RTK/RAS/MAPK) pathway are known to be mutually exclusive. Thus, these mutations likely represent passenger mutations [33,34]. Patient was started on pembrolizumab and had a dramatic response to therapy, with complete resolution of his metastatic disease.

## 4. Discussion

We studied the real-world clinical application of tissue WES/WGS and targeted gene panel sequencing of matched ctDNA. We focused on the effects of timing of genomic analysis, metastatic burden, tumor mutational burden, and systemic treatment on concordance among various cancer types. Consistent with prior studies, our analysis demonstrated a 58% concordance rate at the patient level and a 16% positive concordance rate at the gene level [11,12,13,14,35,36].

TMB is currently a biomarker used to predict response to immunotherapies and targeted agents [37,38,39,40]. It has also been shown to have prognostic significance [41]. Whether ctDNA can replace tissue NGS in a clinical setting to monitor response to immuno-oncology drugs remains to be elucidated, and will need to be confirmed in large prospective studies. Our results showed that the number of ctDNA mutations correlated with increasing TMB only in patients who had received systemic therapy prior to any NGS, and, not in treatment-naïve patients. Thus, ctDNA may be a reliable biomarker for immunotherapy in patients who have received prior systemic therapy, but not, in treatment-naïve patients it may not be a reliable biomarker. However, this would need to be confirmed in a large prospective study.

Another interesting finding in our study was the effect of systemic therapy on concordance. There was no difference in driver somatic alteration detection in relation to receipt of prior systemic treatment seen in matched tissue NGS and ctDNA, suggesting that these concordant driver alterations are truncal mutations in the evolutionary tree of the tumor and are present in every cancer cell. These alterations may reveal therapeutic vulnerabilities within the tumor that can be targeted and therefore be used in drug development [42].

In our study, high concordance was noted in breast cancer patients. Although administration of systemic treatment in this subgroup may have been a confounding factor, we suspect that treatment may have led to the eradication of therapy-sensitive divergent clones and the selection of truncal resistant clones. Recent data suggests that chemotherapy can radically increase the speed of clonal evolution and lead to new malignant and resistant driver clones that form tumor metastases [43]. Therefore, ctDNA analysis following systemic therapy may reveal aggressive driver clones and provide therapeutic targets without the need for repeat biopsy upon progression. One finding that is hard to explain was the higher targetable alteration concordance observed in treatment-naïve patients. Taken together, our data suggest that driver alterations may be a better marker of concordance compared to patient-level or gene-level analysis.

Similar concordance was noted when the time interval between tissue NGS and ctDNA was <90 days and ≥90 days (patient-level: 55% vs. 65% (*p* = 0.43); gene-level: 15% vs. 17% (*p* = 0.50), respectively). In a retrospective analysis, Chae at al. demonstrated that the concordance between paired biopsies less than 90 days apart and paired biopsies greater than 90 days apart was not statistically different [13]. Similarly, in two other studies that used a cutoff of 6 months (≤6 months vs. ≥6 months), the concordance rate was higher with a duration ≤ 6 months for paired biopsies, but results did not reach statistical significance [12,14,44].

Consistent with prior studies [10,45,46], our analysis showed a higher concordance rate for metastatic sites compared to primary tissue sites, although our data did not meet statistical significance. The higher concordance from metastatic biopsy sites might have been due to high tumor burden, higher plasma ctDNA concentration, and greater tumor intra-tumoral and inter-tumoral heterogeneity in the advanced stage tumors. In agreement with previous studies, concordance differences in relation to the number of metastatic sites was not observed across all solid tumors.

As in our previous study, tissue NGS and ctDNA again showed the complementary nature of these two modalities. We found <50% concordance in driver alterations in these tumors. These findings were similar in different tumor types in our analysis. We also noted that the number of potentially actionable alterations was increased when matched ctDNA was obtained in addition to tissue NGS in these patients. These findings are essential, as several clinical trials are underway in solid tumors that are enrolling patients based on ctDNA (NCT03637686, NCT02842203, NCT03145961). Failure to perform ctDNA analysis would deprive these patients of the opportunity to be enrolled in such studies. Therefore, incorporating multiple approaches including ctDNA data and tissue NGS may increase the sensitivity of actionable alterations and consequently expand therapeutic options for patients.

One of the challenges in precision oncology is low drug matching rate with the driver alterations and available tissue agnostic options. In our cohort, molecularly driven therapies were underutilized in our cohort as only 5% (total 3 lung cancer patients: (1) pembrolizumab + crizotinib; (2) pembrolizumab and (3) trametinib + dabrafenib) of patients were treated in this manner (Supplementary Table S3). This finding agrees with other studies that demonstrate the considerable gap in testing and availability of molecularly targeted clinical trials in real-world clinical practice [47,48].

We studied hotspot oncogenic mutations that will have a functional impact as compared to focusing on driver genes mutations, as these hotspot mutations are likely drivers of the malignant process [19]. Therefore, targeting hotspot alterations may be an effective strategy in antineoplastic drug development. In our analysis, *TP53* had by far the most hotspot mutations, without any distinct frequency patterns for enrichment in any subgroup or cancer type. Thus, there did not appear to be selective pressure on any one mutation, which is consistent with previous studies suggesting that *TP53*-inactivating mutations are equivalent in their phenotypic behavior [49]. *KRAS* G12/G13 oncogenic mutations were the most frequent hotspot mutations in our analysis. These were the most recurrent hotspot mutations in all cancer types, except for breast cancer. In looking solely at *KRAS* G12/G13, we found a statistically significant enrichment in concordance when patients were treated with standard-of-care systemic therapies prior to tissue NGS or ctDNA analysis (chemotherapy-treated: 5/5 (100%) vs. chemotherapy-naïve: 3/9 (33%); *p* = 0.031), which suggests that these mutations are likely truncal in the clonal ancestry of tumor evolution and therapeutically viable. Interestingly, in chemotherapy-treated patients, the discordant mutations were only present in tissue NGS. However, this finding may have been a result of the cancer type. Four out of five treated patients with concordant *KRAS* G12 mutations were lung adenocarcinoma, suggesting *KRAS* dependency in every cancer cell, which may suggest *KRAS*-dependent therapeutic vulnerabilities in these tumors. Furthermore, 6/9 *KRAS* G12 mutations in treatment-naïve patients were from either pancreatic or colorectal cancers.

We also described three cases in our clinical practice highlighting the complementary nature of tumor NGS and ctDNA. In two of the cases, ctDNA analysis revealed a targetable alteration that was not initially reported in tissue NGS reports. However, on looking at raw data (BAM) files manually, these alterations were present in the tumor tissue but not reported because these alterations did not pass the threshold of variant allele frequency or copy number cutoff on the prespecified variant caller. This suggests that tissue NGS and ctDNA discordance could partly be due to the technicalities of bioinformatic analysis. Furthermore, our third case was a patient with Lynch syndrome with a high mutational burden. Mutations in the RTK/RAS/MAPK pathway were passenger mutations and have been shown to be mutually exclusive in large-scale projects such as, TCGA and ICGC [33,34]. These examples demonstrate the need for the molecular tumor boards with expertise in molecular pathology and computational biology to recognize these issues impacting patient care.

There were several limitations to our study. The study was performed retrospectively at a single institution. Each tumor subtype had a relatively small number of patients, which precluded a meaningful survival analysis. Thus, findings from this study will need to be confirmed in larger prospective studies. 

## 5. Conclusions

In conclusion, our findings demonstrated that tissue NGS and ctDNA are complementary modalities, with a considerable number of non-overlapping driver and actionable alterations detected by each platform. These modalities have significant therapeutic relevance at present, as several ctDNA-based clinical trials are currently underway. In addition, somatic alterations in the context of TMB and chemotherapies should be considered in future studies. Furthermore, driver and hotspot alterations may be better measures of concordance between ctDNA and tissue NGS, as they represent alterations that have shown to govern tumorigenesis. These findings will need to be confirmed in prospective studies.

## Figures and Tables

**Table 1:** Patient demographics.

Patients Characteristics	Total Number of Patients (*n* = 64)
Median age at diagnosis (years)	66
Sex:	
Male	22 (34.4%)
Female	42 (65.6%)
Type of cancer:	
GI cancer	20 (31.2%)
Lung cancer	19 (29.7%)
Breast cancer	12 (18.7%)
Other malignancies	13 (20.3%)
Median time between tissue biopsy and blood specimen collection	20.5 months
Biopsy site	
Primary tumor	38 (59%)
Metastatic site	26 (41%)
Tumor stage	
Stage III	8 (12.5%)
Stage IV	56 (87.5%)

**Table 2:** Percent concordance (%) at patient and gene level.

Variable	Patient Level (%)	Gene Level								
		*p*-value	All Alterations * (%)	*p*-value	Driver Alterations (%)	*p*-value	Targetable Alterations (%)	*p*-value	Hotspot Alterations (%)	*p*-value
Tumor type										
All tumor types	58		16		9		45		34	
Breast carcinoma	83		20		10		37		36	-
Lung carcinoma	68		22		12		44		43	
GI malignancies	45	-	15	-	12	-	26	-	35	
Tumor mutational burden (mutations/megabase)										
TMB < 2	36	0.017	13	0.40	10	0.72	38	0.27	21	0.013
TMB ≥ 2	69		17		9		20		39	
Chemotherapy status										
Received chemotherapy before testing	78	0.013	21		9		29		43	0.15
Chemotherapy-naïve	43		11	0.014	7	0.31	9.5	0.10	26	
Interval between tissue NGS and ctDNA (days)										
<90	55	0.43	16	0.71	9	0.93	37	0.79	32	0.63
≥90	65		17		9		32		38	
Biopsy site										
Primary site	48		14		10		27		33	1.0
Metastatic site	64	0.20	17	0.50	9	0.71	23	0.74	35	
Number of metastatic lesions										
1 metastatic lesion present	53		15		10		13		39	
>1 metastatic lesion present	55	0.94	17	0.52	9	0.67	27	0.48	32	0.63

* based on gene panel from ctDNA platform used.

**Table 3:** *TP53* hotspots (HS) mutation concordance based on tumor types, systemic therapy status, biopsy site and interval between tissue NGS and ctDNA.

Variable	Total *TP53* HS Mutations	Concordant *TP53* HS Mutations	Percent Concordance of TP53 HS Mutations	Tissue NGS	Tissue NGS (%)	ctDNA	ctDNA (%)
Tumor types							
All tumor types	48	14	29.17%	15	31.25%	19	39.58%
Breast carcinoma	8	2	25.00%	3	37.50%	3	37.50%
Lung carcinoma	16	6	37.50%	3	18.75%	7	43.75%
GI malignancies	13	4	30.77%	4	30.77%	5	38.46%
Other malignancies	11	2	18.18%	5	45.45%	4	36.36%
Chemotherapy status							
Received chemotherapy before testing	22	7	31.82%	6	27.27%	9	40.91%
Chemotherapy-naïve	20	5	25.00%	7	35.00%	8	40.00%
Biopsy site							
Primary site	22	7	31.82%	7	31.82%	8	36.36%
Metastatic site	26	7	26.92%	8	30.77%	11	42.31%
Interval between tissue NGS and ctDNA (days)							
<90	34	11	32.35%	10	29.41%	13	38.24%
≥90	14	3	21.43%	5	35.71%	6	42.86%

**Table 4:** Hotspot (HS) mutation concordance based on tumor types, systemic therapy status, biopsy site and interval between tissue NGS and ctDNA.

Variables	Total HS Mutations	Concordant HS Mutations	Percent Concordance for HS Mutations	Most Frequent HS Mutation	Most Frequent Concordant HS Mutation	Most Frequent Discordant HS Mutation
Tumor type						
All tumor types	91	31	34.07%	*KRAS* G12/G13 (15) *TP53* R175 (4) *KRAS* G12/G13 (6) *KRAS* G12 (7) *KRAS* G12 (2) *TP53* R273 (2)	*KRAS* G12/G13 (9) *TP53* R175 (2) *KRAS* G12/G13 (5) *KRAS* G12 (4) *BRAF* V600 *TP53* E285 *TP53* S241	*KRAS* G12/G13 (6)
Breast carcinoma	14	5	35.71%			
Lung carcinoma	28	12	42.86%			
GI malignancies	31	11	35.48%			
Other malignancies	18	3	16.67%			
Chemotherapy status						
Received chemotherapy before testing	35	15	42.86%	*KRAS* G12 (5)	*KRAS* G12 (5)	*TP53* R248 (2) *TP53* Y220 (2)
Chemotherapy-naïve	46	12	26.09%	*KRAS* G12/G13 (9)	*KRAS* G12 (3)	*KRAS* G12/G13 (6)
Biopsy site						
Primary site	36	12	33.33%	*KRAS* G12 (6)	*KRAS* G12 (4)	*TP53* R213 (3) *TP53* Y220 (3)
Metastatic site	55	19	34.55%	*KRAS* G12/G13 (10)	*KRAS* G12/G13 (5)	*KRAS* G12/G13 (5)
Interval between tissue NGS and ctDNA (days)						
<90	65	21	32.31%	*KRAS* G12/G13 (11)	*KRAS* G12 (5)	*KRAS* G12/G13 (6)
≥90	26	10	38.46%	*KRAS* G12/G13 (4)	*KRAS* G12/G13 (4)	*TP53* R213 (3)

## Supplementary Materials

The following are available online at https://www.mdpi.com/2072-6694/11/9/1399/s1, Figure S1: Scatter plots showing the relationship between tissue tumor mutation burden (TMB, mut/mb) and the number of ctDNA mutations. TMB positively correlated with the number of ctDNA mutations only in breast cancer patients, Figure S2: Venn diagrams showing the number of discordant mutations (Tumor, ctDNA) and concordant mutations among: all mutations found, all driver alterations found, all hotspot alterations found, and all targetable alterations found, Figure S3: Lollipop plots of TP53 hotspot mutations. Figure S3A: All TP53 hotspot mutations identified in our patients, Figure S3B: All concordant TP53 hotspot mutations. No significant consensus hotspot was identified, though they primarily occurred with the DNA binding domain of TP53, Table S1: List of genes from ctDNA targeted gene sequencing, Table S2: Patient demographics, Table S3: Patients who received chemotherapy with concordant gene(s) and mutation(s), Table S4: Patients with documented tissue TMB on sequencing report (lung, breast and GI cancers), Table S5A: List of Concordant Hotspot Mutations, Table S5B: List of Disconcordant Hotspot Mutations.
