# Supplementary material for: Matched Whole-Genome Sequencing (WGS) and Whole-Exome Sequencing (WES) of Tumor Tissue with Circulating Tumor DNA (ctDNA) Analysis: Complementary Modalities in Clinical Practice
Source: Cancers (Basel). 2019 Sep 19;11(9):1399. doi: 10.3390/cancers11091399 (PMC6770276; doi:10.3390/cancers11091399)
Supplement: Supplementary file 1 [file cancers-11-01399-s001.zip › cancers-571910-supplementary/cancers-571910-suppl methods and figures-checked.docx]

Supplementary Materials

Matched Whole-Genome Sequencing (WGS) and Whole-Exome Sequencing (WES) of tumor tissue with Circulating Tumor DNA (ctDNA) Analysis: Complementary Modalities in Clinical Practice

Robin Imperial, Marjan Nazer, Zaheer Ahmed, Audrey E. Kam, Timothy J. Pluard, Waled Bahaj_,_ Mia Levy, Timothy M. Kuzel, Dana M. Hayden Sam G. Pappas, Janakiraman Subramanian and Ashiq Masood

Supplemental Methods

Tissue Whole-Genome Sequencing:

NantOmics Platform

Formalin-fixed paraffin embedded (FFPE) tumor samples (*n* = 49) sent to NantOmics for whole genome sequencing (WGS) were analyzed as previously described [1]. Following DNA extraction, next generation sequencing was carried out using illumina platform. Normal tissue was sequenced at an average read depths at 30× whereas tumor tissue was sequenced at average read depth of approximately 60×. Reference based alignment of both Normal and tumor tissue was carried out using Burrows-Wheeler Aligner [2]. Samblaster was used for duplicate marking [3]. NantOmics Contraster analysis (proprietory) pipeline was used for somatic and germline SNV, insertions and deletions, and identify amplified regions of the tumor genome. Small variants were annotated with base-level PhastCons conservation scores, population allele frequencies from dbSNP (build 142) (https://www.ncbi.nlm.nih.gov/projects/SNP/).

Each small variant predicted to alter the protein sequence of a gene is further analyzed by a proprietary de novo assembly algorithm that realigns all reads surrounding the variant from both tumor and matched-normal samples to increase confidence that the detected somatic or germline variant is real. Variants counts from DNA were calculated form Variant Call Format (VCF) generated files by the bioinformatics pipeline. The true germline and somatic status variable was determined from NantOmics Contraster analysis of tumor and normal DNA sequence data and subsequently used to stratify variant counts. Variables used for variant filtering were downloaded from the dbSNP public database (https://www.ncbi.nlm.nih.gov/projects/SNP/).

Tissue Whole –Exome Sequencing

Tempus Platform

FFPE tumor samples (*n* = 15) sent to Tempus for whole exome sequencing were reviewed by expert pathologist assessment of overall tumor content and percent tumor cellularity as a ratio of tumor to normal nuclei verified specimens met a 40% threshold. Whole exome sequencing were performed similarly as previously described [4]. The amplified target-captured DNA tumor library was sequenced using 2 × 126 bp PE reads to an average unique on-target depth of 150× on an Illumina NovaSeq.

Variant detection, visualization and reporting were performed as previously described [4]. DNA sequencing was aligned to GRCh37 BWA [2]. Duplicate reads were with SAMBlaster [3]. Discordant reads were identified and separated. Following alignment and sorting, SNVs were called. VCF file generated from the variant calling pipeline were processed for computation of read depth and variation in heterozygous germline SNVs between the tumor and normal samples. In addition to SNPEFF algorithm [5], Variant annotations was carried out using multiple criteria including evolutionary models, functional data, clinical data and in-depth literature search. Circular binary segmentation was used for CNV analysis [6]. Tempus calculated Tumor mutation burden by dividing non-synonymous mutations by the megabase size of the exome panel.

Circulating Tumor DNA analysis (ctDNA)

Circulogene Platform

Blood samples (*n* = 14) were sent in EDTA-containing tubes as per Circulogene protocol. Further analysis were carried out as described previously the Circulogene team [7]. Briefly, Ciculating cell free DNA was isolated from plasma samples using CirculoGene’s proprietary cfDNA enrichment and recovery platform (CGT). Each sample was analyzed for the entire 50-gene panel; interrogating about 2,800 mutations. Ultradeep sequencing (3000×–8000×) was performed using Ion Torrent Next Generation Sequencing. The vendor used Ion AmpliSeq Library kit 2.0 and Cancer Hotspot Panel v2 to generate targeted sequencing libraries. VariantCaller 4.2 with high stringency parameters was used for somatic mutation calls. All somatic variants underwent second conformation using GenePool (Station -X). Publicly available databases COSMIC [8], EXac [9] 1000 genome [10], dbGAP (https://www.ncbi.nlm.nih.gov/gap) and GNOMaD [11] was used as a filtering criteria. In addition, stringent quality score was employed. The quality score threshold to report a variant was 10, and any mutation allele frequency under 1% was removed for the results.

Guardant Health Platform

Blood samples were sent to Guardant health (*n* = 49). Blood samples were sent in Streck tubes as per Guardant protocol. ctDNA was collected from these samples and about 30 ng of ctDNA was prepared for next generation sequencing as previously described. The assay clinical sensitivity was reported to be 80.7%, specificity 99.7%, and diagnostic accuracy 99.3% [12].

Briefly, software utilized include: CASAVA (version 1.8.4), the open source BWA-MEM aligner, and a custom read pile-up process that utilizes information encoded by digital-sequencing oligonucleotides to reconstruct the set of unique cfDNA molecules [12]. Custom scripts are then used to (a) remove spurious variants (“noise”) created by sequencing errors, (b) identify all germline single nucleotide polymorphisms (SNPs) and somatic single nucleotide variants (SNVs), and (c) call somatic SNVs, while removing erroneous variants resulting from sequencing errors, DNA damage, strand bias, etc. Germline alterations were not reported by the Guardant Health platform in their clinical reports. Copy number alterations are reported as 1+, 2+, 3+ and greater than 4+.

Supplemental Figure S1


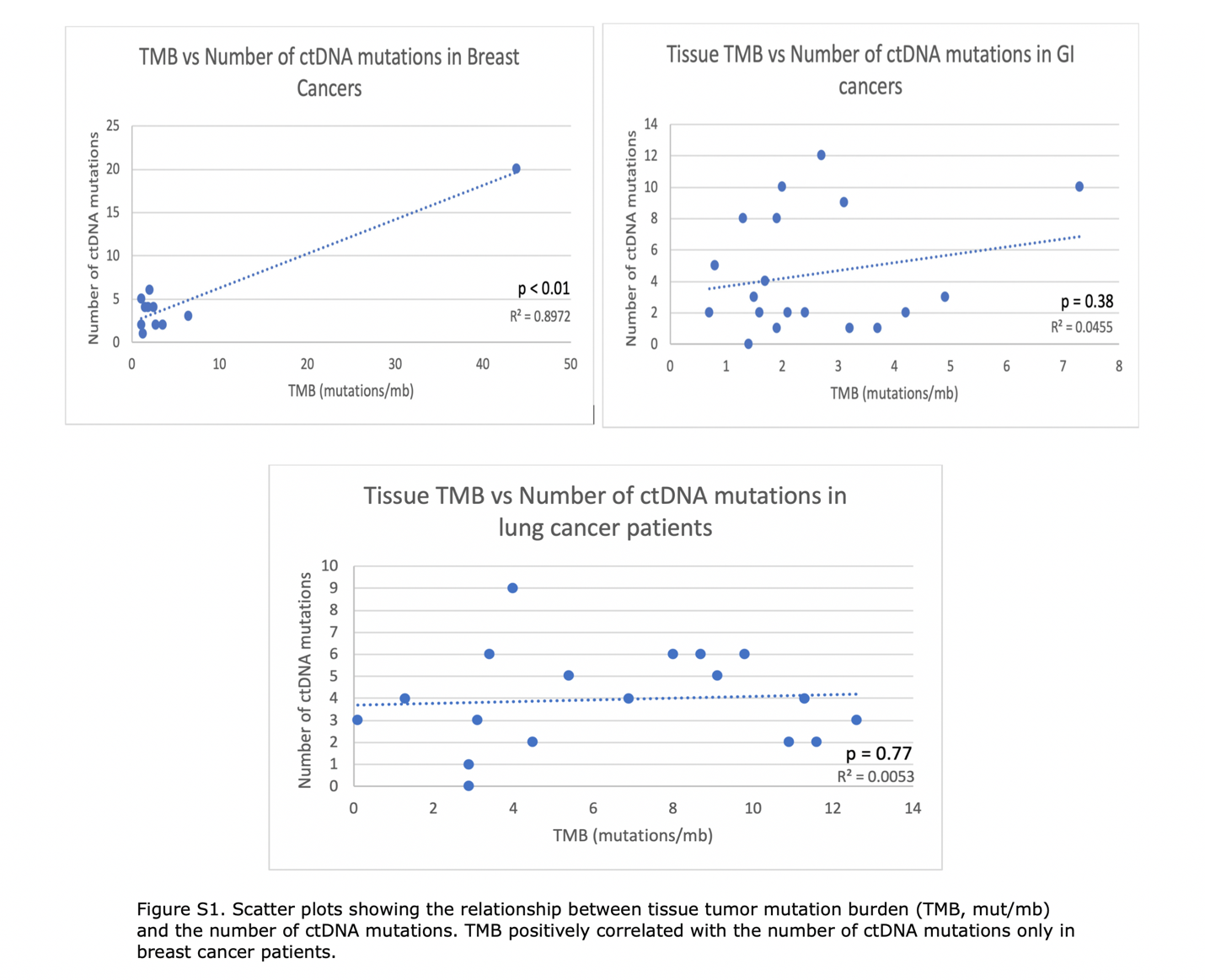


**Figure S1.** Scatter plots showing the relationship between tissue tumor mutation burden (TMB, mut/mb) and the number of ctDNA mutations. TMB positively correlated with the number of ctDNA mutations only in breast cancer patients.

Supplemental Figure S2


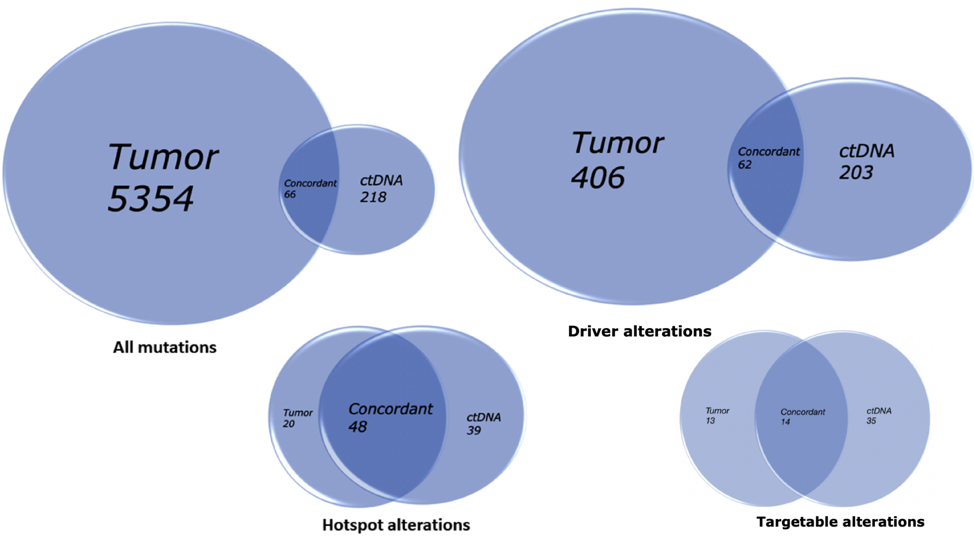


**Figure S2.** Venn diagrams showing the number of discordant mutations (Tumor, ctDNA) and concordant mutations among: all mutations found, all driver alterations found, all hotspot alterations found, and all targetable alterations found.

Supplemental Figure S3


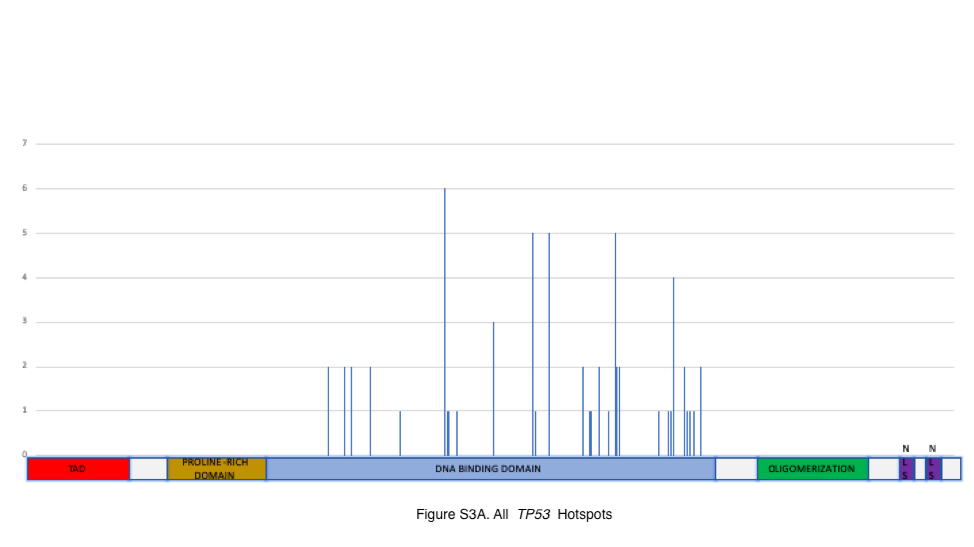


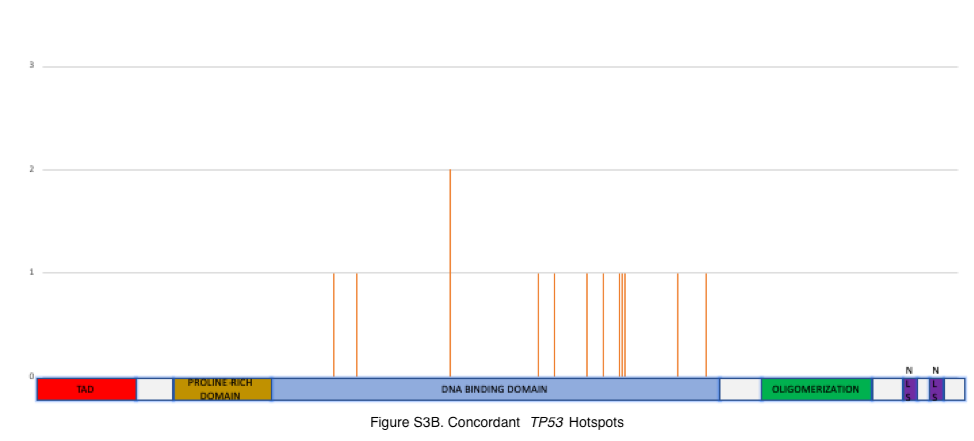


**Figure S3.** Lollipop plots of *TP53* hotspot mutations. Figure S3A: All *TP53* hotspot mutations identified in our patients, Figure S3B: All concordant *TP53* hotspot mutations. No significant consensus hotspot was identified, though they primarily occurred with the DNA binding domain of *TP53*.

Table S1–S5 in a separately Excel file

References

1. Rabizadeh, S.; Garner, C.; Sanborn, J.Z.; Benz, S.C.; Reddy, S.; Soon-Shiong, P. Comprehensive genomic transcriptomic tumor-normal gene panel analysis for enhanced precision in patients with lung cancer. *Oncotarget* **2018,** *9*, 19223–19232.
2. Li, H.; Durbin, R. Fast and accurate short read alignment with Burrows-Wheeler transform. *Bioinformatics* **2009,** *25,* 1754–1760.
3. Faust, G.G.; Hall, I.M. SAMBLASTER: fast duplicate marking and structural variant read extraction. *Bioinformatics* **2014**, *30,* 2503–2505.
4. Beaubier, N.; Tell, R.; Lau, D.; Parsons, J.R.; Bush, S.; Perera, J.; Sorrells, S.; Baker, T.; Chang, A.; Michuda, J. et al. Clinical validation of the tempus xT next-generation targeted oncology sequencing assay. *Oncotarget* **2019**, *10*, 2384–2396.
5. Cingolani, P.; Platts, A.; Wang, L.L.; Coon, M.; Nguyen, T.; Wang, L.; Land, S.J.; Lu, X.; Ruden, D.M. A program for annotating and predicting the effects of single nucleotide polymorphisms, SnpEff. *Fly* **2012**, *6*, 80–92.
6. Olshen, A.B.; Venkatraman, E.S.; Lucito, R.; Wigler, M. Circular binary segmentation for the analysis of array-based DNA copy number data. *Biostatistics* **2004**, *5*, 557–572.
7. Arend, R.C.; Londoño, A.I.; Montgomery, A.M.; Smith, H.J.; Dobbin, Z.C.; Katre, A.A.; Martinez, A.; Yang, E.S.; Alvarez, R.D.; Huh, W.K. et al. Molecular Response to Neoadjuvant Chemotherapy in High-Grade Serous Ovarian Carcinoma. *Mol. Cancer Res*. **2018**, *16*, 813–824.
8. Tate, J.G.; Bamford, S.; Jubb, H.C.; Sondka, Z.; Beare, D.M.; Bindal, N.; Boutselakis, H.; Cole, C.G.; Creatore, C.; Dawson, E. et al. COSMIC: The Catalogue Of Somatic Mutations In Cancer. *Nucleic Acids Res.* **2019**, *47*, D941–947.
9. Lek, M.; Karczewski, K.J.; Minikel, E.V.; Samocha, K.E.; Banks, E.; Fennell, T.; O’Donnell-Luria, A.H.; Ware, J.S.; Hill, A.J.; Cummings, B.B. et al. Analysis of protein-coding genetic variation in 60,706 humans. *Nature* **2016**, *536(7616)*, 285–91.
10. [Auton A](https://www.ncbi.nlm.nih.gov/pubmed/?term=Auton%20A); [Abecasis GR](https://www.ncbi.nlm.nih.gov/pubmed/?term=Abecasis%20GR); [Altshuler DM](https://www.ncbi.nlm.nih.gov/pubmed/?term=Altshuler%20DM); [Durbin RM](https://www.ncbi.nlm.nih.gov/pubmed/?term=Durbin%20RM); [Abecasis GR](https://www.ncbi.nlm.nih.gov/pubmed/?term=Abecasis%20GR); [Bentley DR](https://www.ncbi.nlm.nih.gov/pubmed/?term=Bentley%20DR); [Chakravarti A](https://www.ncbi.nlm.nih.gov/pubmed/?term=Chakravarti%20A); [Clark AG](https://www.ncbi.nlm.nih.gov/pubmed/?term=Clark%20AG); [Donnelly P](https://www.ncbi.nlm.nih.gov/pubmed/?term=Donnelly%20P); [Eichler EE](https://www.ncbi.nlm.nih.gov/pubmed/?term=Eichler%20EE). et al A global reference for human genetic variation. *Nature* **2015**, *526(7571),* 68–74.
11. Karczewski, K.J.; Francioli, L.C.; Tiao, G.; Cummings, B.B.; Alföldi, J.; Wang, Q.; Collins, R.L.; Laricchia, K.M.; Ganna, A.; Birnbaum, D.P. et al. Variation across 141,456 human exomes and genomes reveals the spectrum of loss-of-function intolerance across human protein-coding genes. *BioRxiv* **2019**, Available online: https://www.biorxiv.org/content/early/2019/08/13/531210.
12. Lanman, R.B.; Mortimer, S.A.; Zill, O.A.; Sebisanovic, D.; Lopez, R.; Blau, S.; Collisson, E.A.; Divers, S.G.; Hoon, D.S.; Kopetz, E.S. et al. Analytical and Clinical Validation of a Digital Sequencing Panel for Quantitative, Highly Accurate Evaluation of Cell-Free Circulating Tumor DNA. *PLoS One* **2015,** *10(10):e0140712*.

| 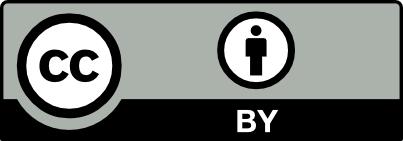 | © 2019 by the authors. Licensee MDPI, Basel, Switzerland. This article is an open access article distributed under the terms and conditions of the Creative Commons Attribution (CC BY) license (http://creativecommons.org/licenses/by/4.0/). |
| --- | --- |
